# Coagulation assessment with thromboelastography during abdominal endovascular aneurysm repair in a patient with hemophilia A

**DOI:** 10.1186/s40981-020-0316-0

**Published:** 2020-02-04

**Authors:** Kazuki Sato, Nobuyuki Katori, Yoshifumi Suga, Shuya Kiyama, Shoichi Uezono

**Affiliations:** Department of Anesthesiology, The Jikei University School of Medicine, 3-25-8 Nishishinbashi, Minato-ku, Tokyo, 105-8461 Japan

**Keywords:** Hemophilia A, Factor VIII, Viscoelastic, Thromboelastography, Heparin, Heparinase, Abdominal aortic aneurysm, Endovascular aortic repair

## Abstract

**Background:**

As both APTT and APTT-based coagulation method cannot distinguish heparin effect from intrinsic coagulation factor deficiency, we implemented thromboelastography (TEG) for the coagulation assessment in a patient with hemophilia A undergoing an endovascular surgery with heparinization.

**Case presentation:**

A 68-year-old male with hemophilia A underwent endovascular aortic repair for abdominal aortic aneurism. TEG results showed recovery of coagulation time (R) in both kaolin assay (CK) and kaolin-heparinase assay (CKH) after factor VIII replacement before heparinization. Against our expectations, R-CKH was slightly prolonged (9.0 min) during heparinization. After the administration of protamine sulfate, R in both assays showed similar values within the normal ranges.

**Conclusions:**

The combination of CK and CKH assays could be useful to estimate factor VIII (FVIII) level when heparin concentration is low or without heparin; however, caution should be necessary for estimation of FVIII level by TEG under the effect of medium- or high-dose heparin.

## Background

Hemophilia A (HA) is a bleeding disorder that is the result of a congenital deficiency of coagulation factor VIII (FVIII). Maintenance of the FVIII level is essential for the perioperative management of patients with HA. Assessment of the FVIII level is also important for adequate FVIII replacement. Activated partial thromboplastin time (APTT) is commonly used as a screening test for FVIII deficiency and for evaluation of FVIII replacement during the perioperative period. An APTT-based, one-stage coagulation method (FVIII:C1) is also broadly used as an FVIII assay for the diagnosis and management of HA. However, these tests may not be reliable for evaluation of the FVIII level under the condition of heparinization because intrinsic coagulation factors other than FVIII are also inhibited by heparin-activated antithrombin. Whole-blood viscoelastic tests, such as thromboelastography (TEG) or rotational thromboelastometry (ROTEM), have recently attracted attention with respect to perioperative coagulation management of a hemophilia patient because these devices can provide multilateral information about coagulation properties rather than a simple intrinsic coagulation test such as APTT or activated clotting time (ACT) [1]. Hence, we implemented the use of TEG for coagulation assessment in a patient with HA who underwent endovascular surgery with heparinization.

## Case presentation

The patient was a 68-year-old male with a height of 158 cm and a body weight of 58 kg who was scheduled for endovascular aortic repair (EVAR) of an abdominal aortic aneurysm. He had a history of persistent bleeding after a tooth extraction at the age of 63 years and received a diagnosis of HA after hematologic examinations. Since that time, he had been treated with FVIII concentrate when he had bleeding owing to a gastric ulcer and when he underwent a colonic polypectomy, although regular concentrate therapy was not implemented because his FVIII level was > 5%. Preoperative coagulation tests indicated a prolonged APTT of 46.8 s and a normal prothrombin time-international normalized ratio of 1.0. His FVIII level was as low as 8%, indicative of mild hemophilia. According to the guidelines for hemostasis strategy for patients with hemophilia without inhibitor published by the Japanese Society on Thrombosis and Hemostasis [2], we planned the following perioperative FVIII replacement protocol: bolus injection of 3000 IU (50 IU/kg) FVIII followed by continuous intravenous infusion at a rate of 240 IU/h (4 IU/kg/h) for 24 h, bolus injection of 6000 IU/day for 5 days after surgery, and bolus injection of 3000 IU/day for the next 3 days.

**Fig. 1:**
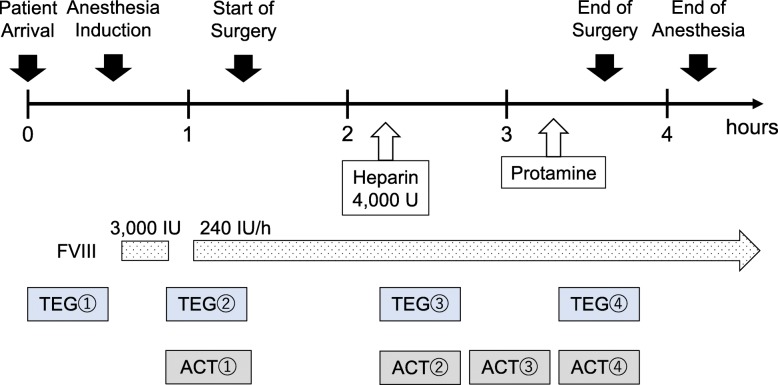
Anesthesia course and coagulation monitoring. TEG was performed at 4 points. ACT was performed simultaneously with TEG and 1 more point during heparinization. ACT, activated clotting time; FVIII, factor VIII; TEG, thromboelastography

Anesthesia was induced with propofol and rocuronium and was maintained with desflurane and continuous infusion of remifentanil. After tracheal intubation and before surgery, we administered a bolus intravenous injection of 3000 IU recombinant FVIII (ADVATE®; Shire, Basingstoke, UK) followed by continuous infusion per the protocol. ACT (normal range, 90–150 s) (Hemochron™ Signature Elite, Instrumentation Laboratory, Bedford, MA, USA) was measured to monitor heparin anticoagulation after the FVIII bolus injection, before unfractionated heparin injection, every 30 minutes during heparinization, and after protamine injection (Fig. 1). Whole-blood coagulation was also examined by TEG (TEG® 6s, Haemonetics®, Braintree, MA, USA) at 4 points: before and after bolus injection of FVIII, after heparin injection, and after protamine injection (Fig. 1). The TEG procedure was performed with a global hemostasis cartridge that includes 4 kinds of assay: CK, CKH, CRT, and CFF. The CK assay is a kaolin-activated assay that examines whole-blood coagulation as a result of activation of intrinsic coagulation factors including FVIII. The CKH assay is a kaolin-activated assay with heparinase which can eliminate effect of heparin in a blood sample that enables to assess the presence of heparin by comparing results between CK and CKH. The CRT assay is a coagulation assay that is activated by both intrinsic and extrinsic activators and allows for rapid evaluation of clot strength. The CFF assay is a functional fibrinogen assay that isolates the fibrin contribution to clot strength and is usually used in conjunction with CK to assess the relative contributions of platelets and fibrinogen to overall clot strength. The reaction time (R) and maximum amplitude (MA) for each assay were evaluated for coagulation management. The R value for CK (reference range, 4.6–9.1 min) is the latency from the start of the assay to initial fibrin formation, which corresponds to APTT. The MA represents the ultimate strength of the clot, which consists of a cross-linked fibrin mesh and activated platelets, although the MA for CFF represents the fibrin mesh strength without the platelet contribution.

**Fig. 2:**
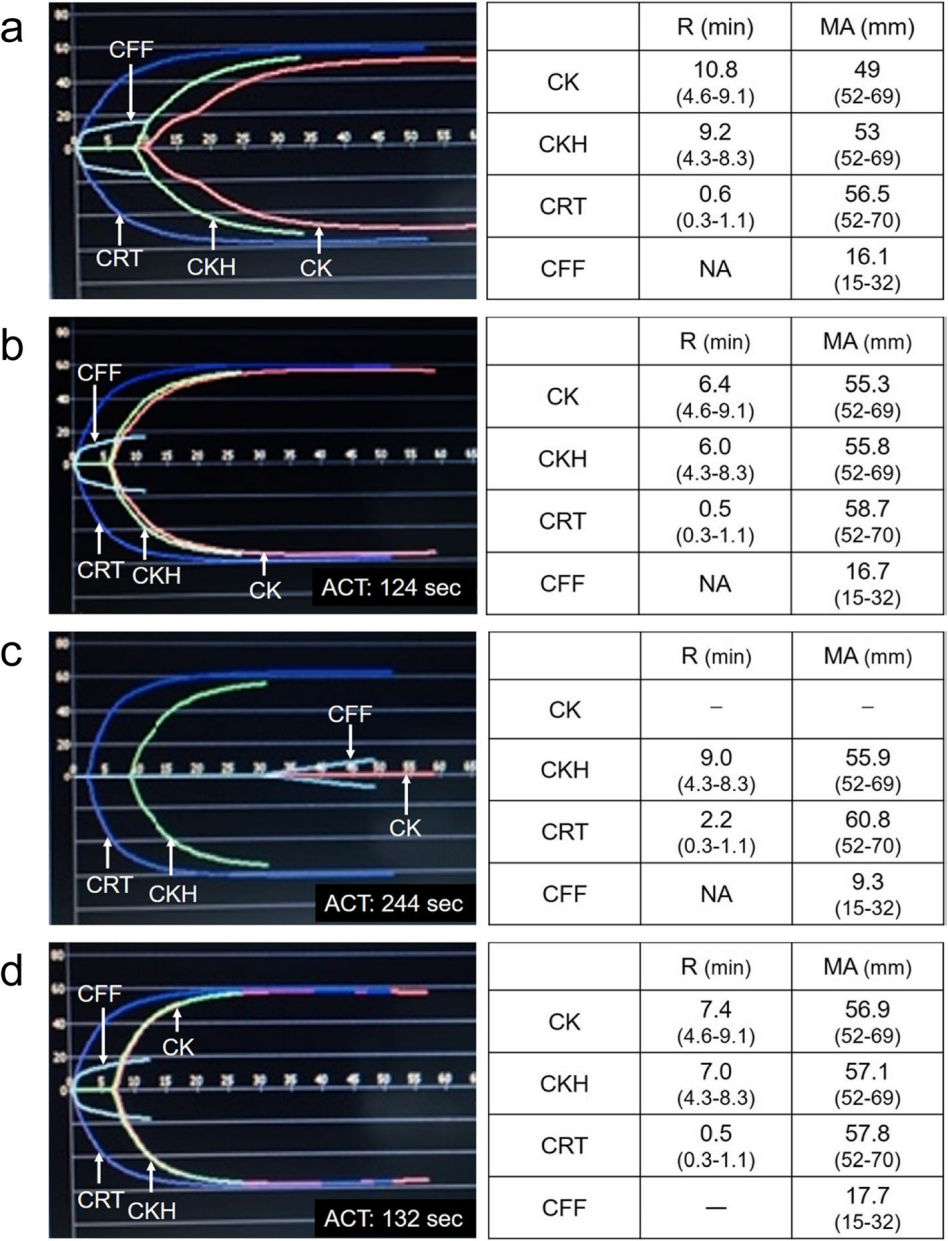
TEG waveforms and numerical values of TEG parameters. **a** TEG data before administration of FVIII concentrate. The R values for both CK and CKH were prolonged, owing to FVIII deficiency. **b** TEG data after FVIII replacement. The R values for both CK and CKH became normal. **c** TEG data after heparin administration. The R value for CK was significantly prolonged, owing to the effect of heparin, although that for CKH was also prolonged by 0.7 min. **d** TEG data after protamine administration. The R values for both CK and CKH were normal. All TEG parameters showed normal values, indicating adequate heparin reversal and preserved levels of coagulation factors including FVIII. Reference ranges for each parameter are indicated within brackets. FVIII, factor VIII; MA, maximum amplitude; R, reaction time; TEG, thromboelastography; NA, not applicable

The results for TEG before administration of FVIII concentrate showed prolonged R values for CK and CKH (10.8 and 9.2 min, respectively) and normal R value for CRT (0.6 min) (Fig. 2a), which indicated retardation of thrombin generation via intrinsic pathway due to FVIII deficiency. After the bolus injection of FVIII concentrate, the R values for CK and CKH were normal (6.4 and 6.0 min, respectively) (Fig. 2b), similarly to the results of normal ACT (124 s), which indicated recovery of thrombin generation as a result of FVIII replacement. After administration of 4000 units of unfractionated heparin, a target range for ACT of 200 to 250 s was achieved. The R for CK (R-CK) was extremely prolonged (unmeasurable), which was presented as a flat line with absent clot initiation in the TEG waveform (Fig. 2c). The R values for CKH and CRT (R-CKH and R-CRT) were also prolonged (9.0 min and 2.2 min, respectively) (Fig. 2c) after heparinization. After the administration of protamine for the reversal of heparin, R-CK, -CKH and -CRT were within normal ranges (7.4, 7.0, and 0.5 min, respectively) (Fig. 2d) as well as normal ACT (132 s). The MA showed normal values for all assays after protamine injection, which indicated that coagulation factor levels, including FVIII and fibrinogen, were maintained at the end of EVAR. The FVIII replacement was continued after surgery, according to the protocol. The postoperative course, including hemostasis, was fair, with no complications.

## Discussion

The maintenance of an adequate level of FVIII is essential for the perioperative management of patients with HA. The recommended level of FVIII to achieve adequate hemostasis during surgery differs depending on the surgical procedure and associated bleeding risk. It is usually considered the risk of perioperative coagulopathy and bleeding is lower in EVAR than in laparotomic aortic repair, but we set a peak target level for FVIII at 100% during surgery, considering the possible risk of conversion from EVAR to laparotomy. Gautier et al. have published a similar case report and described EVAR was advantageous to the standard laparotomic surgery in respect to less need for FVIII concentrate in the perioperative period [3]. But they experienced the development of a hematoma at the femoral artery access site on the first postoperative day.

There are 3 laboratory tests to evaluate FVIII level: FVIII:C1, a 2-stage coagulation method, and a chromogenic assay [4]. Although FVIII:C1 is most broadly used in many hospitals, including ours, because it is simple and is also recommended as a perioperative assay by the guidelines [2], it may underestimate the FVIII level in heparinized blood because some of intrinsic coagulation factors other than FVIII are inhibited by heparin-bound antithrombin [5]. Thus, evaluation of the FVIII level by FVIII:C1 is not accurate during heparinization or in case of insufficient heparin reversal. The APTT or ACT assay could be a surrogate for FVIII:C1, as indicated in the guidelines [2], but these are also affected by heparin. TEG and ROTEM can perform several different assays simultaneously: intrinsic activation, extrinsic activation, intrinsic activation with heparinase, and functional fibrinogen assay. Because total evaluation of these test results could make it comprehensive to understand the cause of perioperative coagulopathy, they have been also examined for the usefulness in the evaluation and treatment of hemophilia patients [6, 7]. Hence, we expected the CKH assay would be useful to estimate the FVIII level even in the presence of heparin owing to heparinase which neutralizes heparin in the blood sample. Unfortunately, our results indicated heparinase used in CKH could not fully neutralize heparin in the blood sample because R-CKH was prolonged during heparinization and returned within the normal range after heparin reversal. Although the blood concentration of heparin in our case was calculated as 1 unit/ml (estimated blood volume, 70 ml/kg), it has been reported heparin activity could be accurately quantified in the range of 0.05 to 0.8 U/ml [8]. CKH would not be suitable for estimation of the FVIII level during heparinization at a concentration higher than 0.8 U/ml, but it could be valid in residual heparin after heparin reversal by protamine. HEPTEM in ROTEM, a similar assay to CKH, has been proved not to be affected by heparin up to 8 U/ml [9]. ROTEM might be better for the estimation of the FVIII level in hemophilic patients during perioperative heparinization.

Maintenance of the FVIII level is also important for appropriate heparin management in cardiovascular surgery. ACT is the mainstay assay for heparin titration during endovascular procedures, but it is also strongly affected by FVIII deficiency, which results in a prolonged value despite the absence of heparin. The prolonged ACT owing to insufficient FVIII replacement can result in underdosing of heparin and inadequate anticoagulation. The use of TEG could facilitate distinguishing between FVIII deficiency and the effect of low-dose heparin (less than 0.8 U/ml) [8], which might contribute to the appropriate heparin management. If the surgical procedure requires medium- or high-dose heparin, TEG could not prove if the FVIII level is adequately maintained during heparinization. In such a situation, the combination of ROTEM and ACT might be more helpful for the appropriate heparin management. However, TEG could be useful for the estimation of the FVIII level at least after heparin reversal, because the TEG results after protamine indicated appropriate heparin reversal without inappropriate FVIII level: there was clinically negligible difference between R-CK and R-CKH, and normal values in all TEG assays and ACT after protamine.

An advantage of implementation of a viscoelastic monitor including TEG is a more comprehensive diagnosis for coagulopathy rather than routine coagulation tests such as APTT or ACT. Although our TEG data after protamine administration indicated normal coagulation properties, including appropriate heparin reversal and adequate FVIII replacement, this advantage would be more important in cardiac surgery [10], in which coagulopathy is more severe owing to the use of cardiopulmonary bypass [5]. The CRT and CFF assays could be performed even in the presence of a certain level of heparin and showed maximally activated clot strength and fibrin mesh strength. In combination with the CK and CKH assays, they could provide more detailed information regarding coagulopathy and aid in making more appropriate decisions regarding coagulation management [11].

We described coagulation assessment by TEG for a patient with HA undergoing EVAR. Both the maintenance of adequate FVIII level and heparinization are important in cardiovascular surgery, and routine coagulation tests, such as APTT and ACT, do not distinguish heparin anticoagulation from FVIII deficiency. Although we expected a kaolin-activated assay with heparinase included in the TEG global hemostasis test could be useful for the estimation of the FVIII level during heparinization, it could neither fully eliminate the effect of heparin nor reflect the FVIII level during heparinization in EVAR. TEG could provide more detailed information about perioperative coagulopathy owing to heparinization or coagulation factor deficiency than APTT or ACT, at least before heparinization or after protamine administration. ROTEM might be more helpful for the estimation of the FVIII level than TEG during perioperative heparinization. Nevertheless, the implementation of viscoelastic monitors that include intrinsic activation assays with and without heparinase, in combination with routine coagulation tests such as APTT or ACT, could be helpful for coagulation management for patients with hemophilia who need heparinization during surgery.

## Data Availability

Not applicable

## Abbreviations

ACT: Activated clotting time
APTT: Activated partial thromboplastin time
EVAR: Endovascular aortic repair
FVIII: Factor VIII
FVIII:C1: One-stage coagulation method for factor VIII assay
HA: Hemophilia A
MA: Maximum amplitude
R: Reaction time
ROTEM: Thromboelastometry
TEG: Thromboelastography
